# Automated Midline Estimation for Symmetry Analysis of Cerebral Hemispheres in FLAIR MRI

**DOI:** 10.3389/fnagi.2021.644137

**Published:** 2021-04-29

**Authors:** Adam Gibicar, Alan R. Moody, April Khademi

**Affiliations:** ^1^Electrical, Computer and Biomedical Engineering Department, Ryerson University, Toronto, ON, Canada; ^2^Department of Medical Imaging, University of Toronto, Toronto, ON, Canada; ^3^Keenan Research Center for Biomedical Science, St. Michael's Hospital, Unity Health Network, Toronto, ON, Canada; ^4^Institute for Biomedical Engineering, Science and Technology, A Partnership Between St. Michael's Hospital and Ryerson University, Toronto, ON, Canada

**Keywords:** FLAIR MRI, image analysis, midline estimation, brain asymmetry, Alzheimer's disease, dementia, texture analysis

## Abstract

To perform brain asymmetry studies in large neuroimaging archives, reliable and automatic detection of the interhemispheric fissure (IF) is needed to first extract the cerebral hemispheres. The detection of the IF is often referred to as mid-sagittal plane estimation, as this plane separates the two cerebral hemispheres. However, traditional planar estimation techniques fail when the IF presents a curvature caused by existing pathology or a natural phenomenon known as brain torque. As a result, midline estimates can be inaccurate. In this study, a fully unsupervised midline estimation technique is proposed that is comprised of three main stages: head angle correction, control point estimation and midline generation. The control points are estimated using a combination of intensity, texture, gradient, and symmetry-based features. As shown, the proposed method automatically adapts to IF curvature, is applied on a slice-to-slice basis for more accurate results and also provides accurate delineation of the midline in the septum pellucidum, which is a source of failure for traditional approaches. The method is compared to two state-of-the-art methods for midline estimation and is validated using 75 imaging volumes (~3,000 imaging slices) acquired from 38 centers of subjects with dementia and vascular disease. The proposed method yields the lowest average error across all metrics: Hausdorff distance (HD) was 0.32 ± 0.23, mean absolute difference (MAD) was 1.10 ± 0.38 mm and volume difference was 7.52 ± 5.40 and 5.35 ± 3.97 ml, for left and right hemispheres, respectively. Using the proposed method, the midline was extracted for 5,360 volumes (~275K images) from 83 centers worldwide, acquired by GE, Siemens and Philips scanners. An asymmetry index was proposed that automatically detected outlier segmentations (which were <1% of the total dataset). Using the extracted hemispheres, hemispheric asymmetry texture biomarkers of the normal-appearing brain matter (NABM) were analyzed in a dementia cohort, and significant differences in biomarker means were found across SCI and MCI and SCI and AD.

## 1. Introduction

Half a million Canadians are living with dementia and 25,000 new cases are diagnosed per year. By 2031, this is expected to increase by 66% to more than 1 million Canadians, carrying a $16.6B cost per year to care for them (Manuel et al., 2016). There has been numerous failed clinical trials targeting beta-amyloid plaques with no resulting cure or disease modifying treatment (Yiannopoulou et al., 2019). Determining whom to treat and when requires reliable and clinically valid biomarkers that identify disease early and characterize disease trajectories.

Computer generated biomarkers measured from magnetic resonance imaging (MRI) can be used to stratify patients, identify new targets, detect disease early and monitor disease progression. Clinically, biomarkers can be used to determine optimal intervention points. There have been numerous research efforts over the years to identify reproducible biomarkers from neurological MRI that are related to cognition including GM volumes (Taki et al., 2011), WM volumes and integrity (Pievani et al., 2010) as well as white matter lesions (WML) (Meng et al., 2017). There is also growing evidence that neurodegenerative diseases may affect cerebral symmetry in MRI (Toga and Thompson, 2003; Feis et al., 2019). Studies have demonstrated that patients with more advanced dementia have larger cortical volume asymmetries (Rombouts et al., 2000; Karas et al., 2004; Kim et al., 2012). More recently, there has been gaining interest in identifying microstructural asymmetry biomarkers as a mechanism to understanding the underlying diffusion and structural integrity of underlying WM and GM. In Derflinger et al. (2011) and Yang et al. (2017), through diffusion tensor tractography and voxel-based morphometry (VBM), microstructrual symmetry analysis revealed asymmetric topological organization in WM networks and asymmetric GM loss in patients with AD. Due to the increasing prevalence of neurodegeneration and dementia diseases and their interaction with brain asymmetries, this work focuses on tools that enable automated cerebral hemisphere analysis.

Although many sequences are available to study neurodegenerative diseases, Fluid-Attenuated Inversion Recovery (FLAIR) MRI is a preferred sequence for analyzing vascular disease (Alber et al., 2019) which is the second leading risk factor for dementia (Román, 2002). This is because the cerebrospinal fluid (CSF) signal is nulled in FLAIR MRI which highlights ischemic and demylinating pathology with high intensities (Alber et al., 2019). Automated algorithms that quantify brain asymmetry in FLAIR MRI can facilitate large scale analysis of retrospective databases to identify patterns that aid in understanding the etiology and pathogenesis of neurodegeneration, dementia and vascular disease. Performing symmetry analysis on FLAIR-MRI can aid in the identification of vascular risk factors and can be used to develop new therapies (Frey et al., 2019). Clinically, since FLAIR MRI are routinely acquired, automated asymmetry analysis tools can be integrated into clinical workflows to characterize vascular and neurodegnerative diseases in real-time.

To perform brain asymmetry analysis in FLAIR MRI, the cerebral hemispheres must be extracted. This can be completed by detecting the interhemispheric fissure (IF), which corresponds to the midsagittal plane (MSP) that separates cerebral hemispheres. Normally, the human brain exhibits an approximate bilateral symmetry with respect to the IF. A natural phenomenon known as brain torque can cause asymmetries of the IF. Brain torque results in clear visible bending along the entire fissure, more prominently in the occipital lobe (Xiang et al., 2019). It is assumed to from a lateralized gradient of embryological development of the human brain (Xiang et al., 2019). Traditional techniques for midline plane estimation do not account for IF curvature which results in a poor separation of cerebral hemispheres (Stegmann et al., 2005). Therefore, for optimal separation of cerebral hemispheres, IF curvature should be detected.

Midline plane estimation algorithms in MRI are classified into two types: symmetry-based and shape-based. Symmetry-based approaches, also known as content-based, optimize a symmetry metric computed between candidate cerebral hemispheres until the optimal hemispheric separation is found (Ferrari et al., 2016). Ruppert et al. (2011) proposed an MSP algorithm based on bilateral symmetry maximization. In this approach, symmetry is quantified using edge features and the optimal plane is sought through maximizing the correlation between the original image, and a flipped copy with respect to a candidate plane (Ruppert et al., 2011). Shape-based algorithms make use of an initial estimation of the IF and use it as a landmark to fit a plane from points that lie in the IF region (Ferrari et al., 2016). A classic shape-based algorithm by Brummer (1991), implemented a three-dimensional variant of the Hough transform to detect lines in each coronal slice and computed the MSP using interpolation.

In traditional midline estimation algorithms, the result is planar and three dimensional, meaning each axial slice contains the same midline estimation. Although promising, these methods may not be optimal in the presence of midline curvature or shift, and could have higher error since the amount of IF curvature can vary slice to slice. Midline plane estimation can be used as a preprocessing step for more accurate midline detection, but any error in this step is propagated to later phases of the algorithm. Attempts to improve on previous approaches to account for IF curvature include work from Stegmann et al. (2005), which defines the curved MSP as the mid-sagittal surface (MSS). They proposed a MSS estimation algorithm that fits a thin-plate spline to the brain data using a robust least median of squares estimator (Stegmann et al., 2005). This method results in more accurate separation of cerebral hemispheres but is more computationally expensive (Stegmann et al., 2005). While MSS methods are better to handle IF curvature, the septum pellucidum, a membrane separating the lateral ventricles, can causes issues for MSS-based approaches.

To overcome the challenges of traditional midline methods, this work proposes a novel and robust mid-sagittal surface (MSS) estimator that accounts for IF curvature in multicenter FLAIR datasets. It does not require a MSP pre-processing step, is completely unsupervised and estimates the midline on a per-slice basis. The issue of curvature in the IF is addressed through local optimization of control points (spatial coordinates) determined by extracting features in the vicinity of the IF. Control points are estimated based on local contrast, texture, intensity, and symmetry features at the same time, and post-processing is completed to ensure the features are robust across the volume and in septum pellucidum region. Polynomial fitting is utilized to detect the midline from the control points and the brain can be separated into the left and right hemispheres. To evaluate performance of the proposed MSS algorithm, a series of validation metrics are used on a midline validation dataset comprised of 75 volumes (~3,000 image slices). Data was sampled from three databases of vascular disease (CAIN) (Tardif et al., 2013), Alzheimer's disease (ADNI) (Aisen et al., 2015) and dementia (CCNA) (Mohaddes et al., 2018; Chertkow et al., 2019; Duchesne et al., 2019) from three different scanner vendors representing a diverse multi-center, multi-disease FLAIR MRI database for testing across a wide variety of imaging characteristics. The proposed MSS method was compared to two works that perform midline estimation in the literature. The first is a traditional, planar approach by Bergo et al. (2008) which was re-implemented for FLAIR MRI in this work. The second method is by Kuijf et al. (2014), which addresses curvature of the IF and is available as open source software. Through the combination of shape- and symmetry-based approaches, the proposed method accurately detects midlines over all images, including regions with IF curvature or septum pellucidum.

In addition to a novel midline detection method, two other innovations are presented. First is a method to automatically gauge midline separation performance in large datasets without ground truths. It is based on an asymmetry index (AI), which measures the volume difference across hemispheres and z-scores are used to determine segmentation outliers. A total of 5,360 volumes (~275,000 image slices) from 86 centers from CAIN, ADNI, and CCNA databases were used to test the outlier detection approach. Midlines were extracted over the entire dataset and AI outliers were flagged and visually inspected. Out of 5,360 volumes, only 53 were detected as outliers. This can be applied on retrospective large scale studies or real-time on prospective datasets. Lastly, as a proof of concept, microstructural asymmetry biomarkers from the normal-appearing brain matter (NABM) are extracted and compared across subjects in CCNA, for subjects with AD, mild cognitive impairment (MCI) and subjective cognitive impairment (SCI).

## 2. Materials and Methods

In this section, we will describe the data used in this analysis, along with the methods used to extract the midline, and the experimental design.

### 2.1. Data and MRI

The FLAIR MRI data used in this study is from 3 datasets: the Alzheimer's Disease Neuroimaging Initiative (ADNI) (Aisen et al., 2015), the Canadian Atherosclerosis Imaging Network (CAIN) (Tardif et al., 2013) and the Canadian Consortium on Neurodegeneration in Aging (CCNA) (Mohaddes et al., 2018; Chertkow et al., 2019; Duchesne et al., 2019). ADNI is an open source dementia dataset with longitudinal imaging data from 889 subjects, acquired at 58 imaging centers, resulting in a total of 4,264 FLAIR image volumes for analysis (ADNI-2 cohort). This dataset contains subjects within the following disease classifications: Normal, Early Mild Cognitive Impairment (EMCI), Late Mild Cognitive Impairment (LMCI), Subjective Memory Concerns (SMC), and AD (Aisen et al., 2015). The CAIN dataset is from a pan-Canadian clinical study that investigates cerebrovascular disease (CVD). The database contains data from 386 subjects with cerebrovascular risk factors and a varying numbers of follow-up scans, from eight centers, yielding a total of 922 FLAIR imaging volumes. The CCNA dataset is a Canada-wide initiative to strengthen Canadian research on Alzheimer's disease (AD) and related neurodegenerative disorders (NDDs) (Mohaddes et al., 2018). Currently, the FLAIR data from the study contains imaging volumes for 380 subjects, acquired at 20 imaging centers. The FLAIR MRI datasets were acquired on scanners from three vendors (GE, Siemens, and Philips), from over 80 institutions worldwide with variable acquisition parameters. All three data sets are multi-center and multi-vendor FLAIR-MRI scans, representing a diverse dataset of one of the largest FLAIR MRI databases analyzed in the literature. More information on FLAIR acquisition parameters and subject demographics for ADNI, CAIN and CCNA can be found in Table 1. This dataset in its entirety is used for outlier rejection to find poor quality hemispheric segmentations automatically.

**Table 1:** Summary of ADNI, CAIN, and CCNA datasets.

	**Dataset Information**					
**Database**	**No. volumes**	**No. images**	**No. patients**	**No. centers**	**Age (years)**	**M/F (%)**
ADNI	4,264	213K	889	58	73 ± 7	53/47
CAIN	922	46K	386	8	74 ± 8	58/42
CCNA	380	19K	380	20	73 ± 7	56/44
	**Acquisition Parameters**					
**Database**	**Mag Field (T)**	**TR (ms)**	**TE (ms)**	**TI (ms)**	**Pixel Spacing (mm)**	**Slice Thickness (mm)**
ADNI	1.5-3	6,000–11,900	90–192	2,000–2,800	0.7812–1	2–6
CAIN	3	8,000–11,000	117–150	2,200–2,800	0.4285–1	3–5
CCNA	3	9,000–9,840	111–148	2,250–2,500	0.9375	3

To evaluate the performance of the proposed midline estimation algorithm, a midline validation dataset was created by sampling 75 FLAIR MRI volumes (~3,000 images) from all three datasets. In total, there were 25 volumes from CAIN, 25 from CCNA and 25 from ADNI. The data sampling strategy included stratification across centers and scanners where possible, resulting in images from 38 different centers with 27% Philips, 23% GE, and 50% Siemens scans. When stratifying by both center and scanner in CCNA, it resulted in more Siemens volumes being sampled, since 288 of the 380 volumes in CCNA are acquired with Siemens scanners. In ADNI, the diagnosis labels are distributed by varying level of cognition. Of the 25 sampled volumes, five were cognitively normal (CN), nine early mild cognitive impairment (EMCI), six late cognitive impairment (LMCI), and five AD cases. In CCNA, the 25 validation volumes included various dementia cases which are: six AD cases, nine MCI, two SCI, two Parkinson's disease (PD) cases, one Lewey Body disease (LBD), one PD-MCI, and four vascular mild cognitive impairment (V-MCI) cases. In CAIN, clinical diagnosis labels are not available. This represents a diverse set of dementia related diseases in the validation set. Details regarding the midline validation dataset can be found in Table 2. Ground truth midlines were generated by a biomedical student trained by a radiologist using ITKSnap. Midlines were delineated along the interhemispheric fissure by following the region of CSF for all slices (Yushkevich et al., 2006). When there was a shift in the midline for a given slice, the curvature was carefully delineated. To examine automated symmetry analysis using local texture analysis, the CCNA dataset is used. CCNA has diagnostic labels for each subject. All subjects were included that had the specific diagnostic label of interest, except for scans from a specific center that contained high bias field artifacts. In this work, the Alzheimer's Disease (AD), Mild Cognitive Impairment (MCI), and Subjective Cognitive Impairment (SCI) diagnostic labels are used (Mohaddes et al., 2018). MCI describes individuals with memory impairment greater than what would be expected of their age. It is a clinical state between normal cognitive changes due to age and early stages of AD (Petersen, 2000). SCI describes individuals with self-experienced persistent decline in cognitive ability, but achieve normal cognitive scores (Jessen et al., 2014). In total, there are 50 SCI, 98 MCI, and 43 AD cases from CCNA used in this analysis.

**Table 2:** Data summary of sampled ground truth volumes.

**Database**	**Disease**	**No. volumes**	**No. centers**	**GE/** **Philips/** **Siemens**	**Age (years)**	**M/F (%)**
ADNI	Alzheimer's	25	22	6/11/8	73 ± 6	52/48
CAIN	Vascular	25	8	9/8/8	71 ± 6	64/36
CCNA	Dementia	25	8	2/4/19	73 ± 5	60/40

### 2.2. Midsaggital Surface Estimation

**Figure 1:**
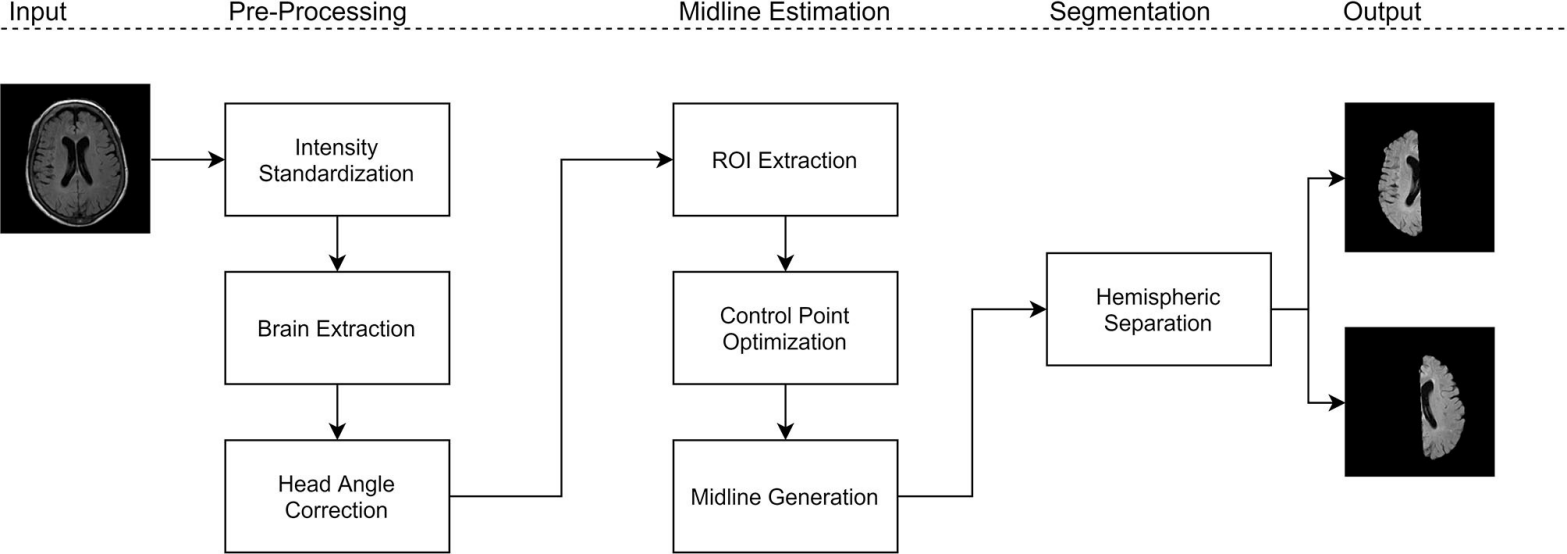
Experimental design for the proposed method.

The proposed work is a midsagittal surface (MSS) estimation algorithm designed to extract cerebral hemispheres, which enables clinical applications through brain asymmetry studies. First, intensity standardization is used to normalize the range of intensities and reduce variability in multicenter data. Next, brain extraction is performed to remove non-cerebral tissue which permits for robust analysis of the hemispheres. On the intensity standardized and brain extracted data, the midline is extracted using the proposed method. Using the extracted midline for each slice, the brain is separated into cerebral hemispheres that can be analyzed for asymmetry. Figure 1 shows block diagram for the proposed framework.

#### 2.2.1. Pre-processing

Before midline estimation, pre-processing steps are utilized to improve robustness of the algorithm. First, intensity standardization is performed to remove variability caused from the multi-center (MC) effect (Reiche et al., 2019). The standardization algorithm is a framework developed for multi-institutional FLAIR MRI datasets by Reiche et al. (2019) that reduces intensity variability caused by different scanning devices. Denoising, bias field reduction and background subtraction is applied first. Following this, intensity standardization is facilitated through the combination of normalization, scaling, and histogram peak alignment. The standardization pipeline is able to preserve different pathologies, such as white matter lesions (WMLs) (Reiche et al., 2019). This technique is utilized to ensure the same features can be used and the interhemispheric fissure can reliably be extracted in multicenter datasets. Following intensity standardization, brain extraction (BE) is performed on the dataset to ensure midline estimation and symmetry analysis is performed only on cerebral tissues. The brain extraction method is based on a U-Net for intracranial volume (ICV) for FLAIR MRI (Khademi et al., 2019; DiGregorio et al., 2021).

#### 2.2.2. Midline Estimation

Using intensity standardized and brain extracted volumes, midline estimation is performed. The proposed midline estimation method can be divided into three main components: (1) head angle correction, (2) control point optimization, and (3) midline generation. Using the estimated midline for each slice, the cerebral hemispheres can be extracted across the volume.

**Head angle correction**. Head angle correction is employed in this work as a preprocessing step to align the head with the longitudinal axis to improve robustness of midline estimation. The method is inspired by the midline plane estimation algorithm proposed by Liu et al. (2001) which used the head angle to estimate the plane of the midline. To estimate the head angle, the concept of bilateral symmetry is used, where a bilaterally symmetric image *S*_*i*_ about its symmetry axis produces an image *R*_*i*_ that is approximately identical to *S*_*i*_ (Liu et al., 2001). Thus, the head angle is estimated by maximizing the cross correlation between the original image, *S*_*i*_ and its reflected image, *R*_*i*_ using the following steps:

1. Reflect *S*_*i*_ over the vertical axis.
2. For each angle θ, rotate *R*_*i*_ by 2θ and compute the cross correlation of *R*_*i*_ and *S*_*i*_.
3. Find the angle that corresponds to the maximum cross correlation value.
4. Use the optimized θ to rotate *S*_*i*_ and correct for head angle orientation.

**Figure 2:**
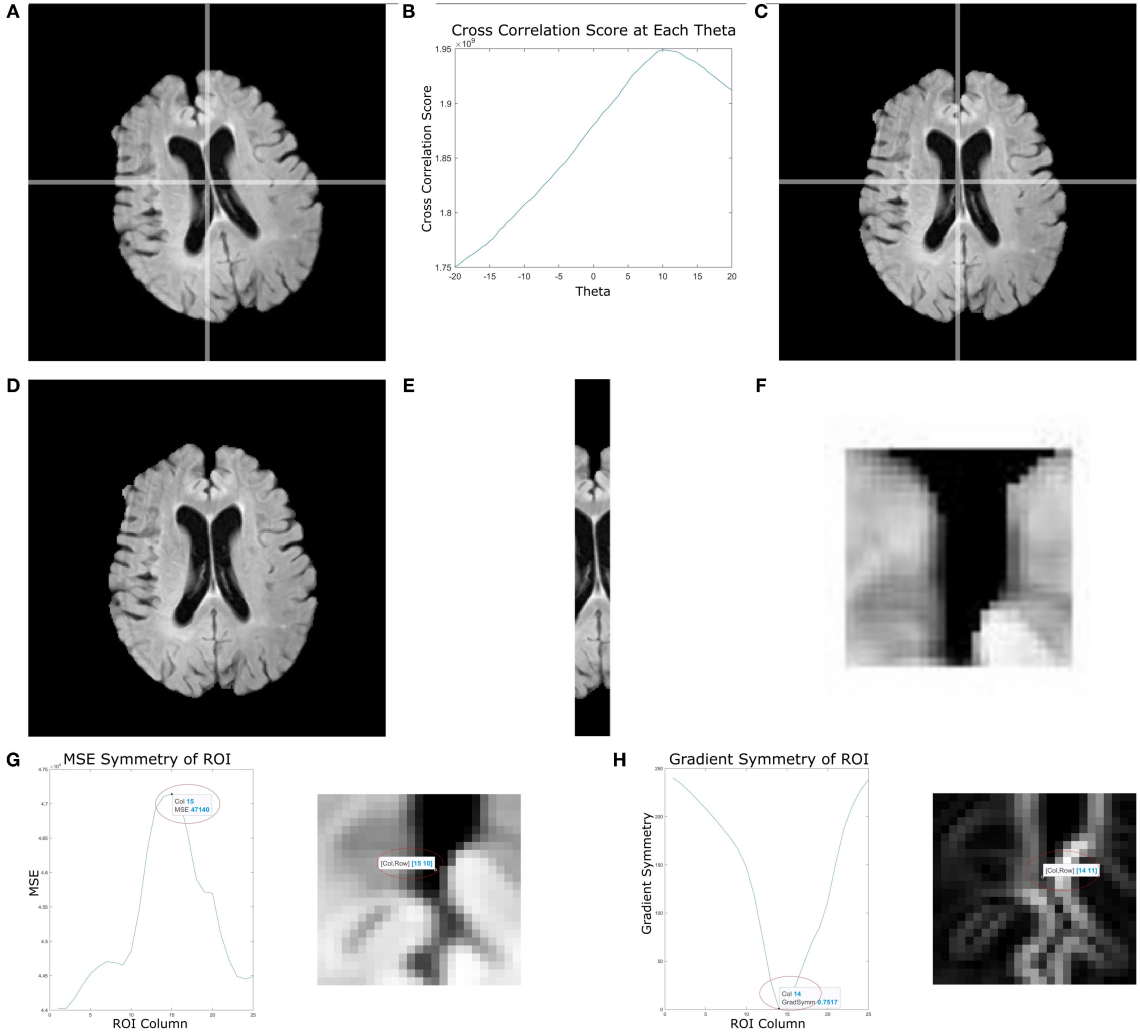
This figure depicts multiple stages of the proposed midline estimation. **(A–C)** Head angle correction step where **(A)** is the original image, **(B)** is the cross correlation values for each θ, and **(C)** the image with corrected head angle. **(D–F)** ROI extraction where **(D)** is the pre-processed image, **(E)** is the ROI 2 cm around the middle column, **(F)** is the 2 by 2 cm window starting from the first non-zero row. Lastly, **(G,H)** show symmetry feature optimization, where **(G)** is the mean squared error symmetry and **(H)** is for gradient-based symmetry.

In this implementation, the angle is estimated based on the middle slice of the volume and θ values ranged from −20 to 20°, with 0.5° increments to account for right and left angled brains. Figures 2A–C shows the head angle correction algorithm for an example slice. Figure 2A shows the original image, and B shows the cross correlation for various angles θ obtained by reflecting the original image, rotating it and computing the cross correlation. As can be seen the maximum cross correlation value is at θ = 10°. Using this angle, the original image is rotated, and shown in Figure 2C, which shows the head is aligned with the longitudinal plane.

**Control point estimation**. Once the head angle is corrected, control point estimation is performed. Control points are locations estimated along the interhemispheric fissure found using a combination of symmetry- and shape-based approaches to characterize CSF in the midline. The midline is estimated based on these control points along the fissure.

Control point estimation is performed for cerebral slices only, as these are the most important slices for hemispheric analysis. Moreover, the interhemispheric fissure (IF) is well-defined in the cerebral slices, when compared to slices containing the cerebellum. The method begins by automatically selecting a rectangular ROI containing the IF which is found by extracting a 2 cm region centered around the middle column of the image that extends the length of the image. Given that head angle correction has oriented the head along the longitude plane, this ROI contains the midsagittal plane. A window size of 2 cm ensures both the brain tissue surrounding the IF and the CSF inside the IF are contained within the ROI. An example of the extracted 2 cm region can be seen in Figures 2D–F, which shows an original slice and the corresponding 2 cm region that includes the interhemispheric fissure. Beginning from the first non-zero row of the image, each 2 × 2 cm ROI are extracted and several features are computed to estimate the control points along the midline.

The features used in this work are focused on simultaneously describing the local intensity, gradient, texture and symmetry information of the CSF region within the IF (as compared to the brain). This differs from previous approaches that rely mainly on a single feature. For example, in Jayasuriya and Liew (2012), the authors use intensity profiling for estimating the midline plane. In Bergo et al. (2008) mean intensity was used to search for an initial candidate plane and Chen et al. (2015) used local intensity and gradient symmetry to estimate midline shift of the IF in patients with cerebral glioma. The use of several descriptors to optimize control points yields a more robust and informative encoding of CSF properties within the IF. Moreover, compared to previous works that were developed for other sequences, such as T1 (Jayasuriya and Liew, 2012; Rehman and Lee, 2018), this work focuses on fine-tuning the features specifically for FLAIR MRI. Due to intensity standardization, the extracted intensity, gradient and texture features are consistent across slices and tissues. Depending on the feature, the minimum or maximum value is used to estimate the control point for the particular feature. All features are combined (described later) to arrive at the final control point estimation for that ROI.

In FLAIR MRI, CSF appears as low intensity and therefore intensity is a discriminative feature that is explored. The intensity features used are the intensity sum, energy, root-mean-square (RMS), and cumulative energy. To improve control point estimation, gradient and texture features are also included. The gradient magnitude enhances edges within the ROI around the midline and the IF is localized between edge peaks with a low gradient value. See Figure 2H which shows the original ROI and the corresponding gradient image. There are large edge magnitudes localizing the brain and CSF tissue boundaries, and low edge strength between these edges. To describe texture differences between the IF and surrounding brain tissue, the Gabor transform is used. Texture features have been commonly used in medical image analysis for pattern recognition, segmentation and classification (Castellano et al., 2004). The Gabor filter bank deployed in Roslan and Jamil (2012) for skull stripped T1, T2, and FLAIR MRI brain images is used. For each ROI, the Gabor energy is measured and the maximum energy is used to detect the midline (indicates more homogeneity, i.e., the CSF).

The final set of descriptors used to find control points along the IF are symmetry features. The proposed symmetry features used are mean squared error (MSE) and mean gradient symmetry (MGS). For each ROI, every column is used as a candidate midline to separate hemispheres. The MSE symmetry feature is found by computing the difference in intensities across the hemispheres extracted using the candidate. Hemispheric separation results in two image of the same size as the original, with the left and right sides zeroed out for the right and left hemisphere, respectively. When computing the MSE, the maximum error corresponds to the candidate column that yields the best separation of hemispheres, since the intensities of one hemisphere occur where it is zero valued in the other. For the MGS, the gradient image is used, and the difference in gradient magnitude across the candidate hemispheres is computed. The MGS should be minimum when the correct midline has been selected. An ROI and the corresponding symmetry features is shown in Figures 2G,H where the x-axis represents the column number of the image (ROI). In Figures 2G,H, the detected control point for MSE and MGS occurs at the column that optimally separates the hemispheres and is retained as a candidate control point in the IF.

For each feature calculated, an estimated control point is obtained for the location of the IF in the current ROI. Depending on the feature, either the maximum or minimum value is retained and these are used to find the spatial coordinate of the candidate control point for that feature. To combine all features (intensity, texture, gradient and symmetry) and obtain a single control point per ROI that best describes the IF, the median of the estimated control points for all features is taken. The median is used to ensure outliers do not negatively affect analysis. This analysis is repeated on all ROI (every 2 cm) resulting in a vector of control points along the interhemispheric fissure for every slice. Each slice has its own control points (slice-slice refinement) to ensure midline curvature is detected if present in any slices.

There are two additional tests that are performed to maximize robustness. The first is completed by inspecting control points or the same ROI across the volume (i.e., same ROI over different slices). The estimated control points for this ROI across the volume is treated as a distribution and any extreme points (i.e., three scaled median absolute deviations (MAD) from the median) are flagged. These flagged (outlier) control points are then replaced by a linearly interpolated control point value based on the control points for the same ROI in the two neighboring slices. A second automated test is utilized to ensure the method is robust in the septum pellucidum region between ventricles. These bright regions can cause the midline to be incorrectly estimated into the ventricles (which are dark) instead of along the midline. To mitigate this possibility, a binary mask of the CSF is generated based on thresholding the intensity standardized ROI. If CSF makes up more than 50% of the ROI, then the image intensity is inverted which transforms the CSF inside ventricles to bright intensities, and the septum pellucidum to appear dark, which enables the control point estimation to behave as in CSF-filled IF regions.

**Midline generation**. Given a vector of control points for every slice, the midline is generated using a shape-preserving piece-wise cubic interpolation function, which computes the best fit line along the IF given the control points for that slice. To maintain curvature and smoothness in the estimated midline, a third order polynomial fitting is completed using least squares over the interpolated midline. A third order polynomial is chosen to balance between smoothness and overfitting.

**Hemispheric separation**. Given the estimated midlines for every slice, hemispheric separation is performed. The midline coordinates are used to determine which pixels reside in the left or right hemisphere and binary masks for the corresponding regions are generated for each slice in the imaging volume. Using the extracted cerebral hemispheres, asymmetry biomarkers can be analyzed.

### 2.3. Midline Validation

To validate the proposed midline estimation algorithm, this work will be evaluated over a set of ground truth images and will be compared to two other methods for midline estimation. Performance is quantified with three different validation metrics: (1) mean Hausdorff distance, (2) mean absolute distance, and (3) mean volume-difference, which all compare the automated midline to the ground truth delineations.

The first method that is compared to is from Bergo et al. (2008), and based on the detection of the midsagittal plane (MSP) for T1 MRI. In this work, we have re-implemented the method for multicenter FLAIR MRI based on the intensity standardized imaging data. The method is composed of two main stages to search for the plane that contains the most CSF (excluding ventricles). In this method, the brain is automatically segmented and CSF is removed prior to midline estimation. The IF is located by searching for a candidate plane that intersects the brain masks while minimizing the mean voxel intensity (i.e., CSF) (Bergo et al., 2008). Using the candidate plane, a set of rotations and translations are applied to fine-tune the results by minimize the intensities in these transformed candidate planes. If none of the transformations lead to a plane with a lower intensity score, the current plane is taken as the MSP and the algorithm stops (Bergo et al., 2008). The authors report errors due to irregular, non-planar fissures (Bergo et al., 2008).

The second method is proposed by Kuijf et al. This method is publicly available via GitHub (Kuijf et al., 2014) and is one of the methods that addresses the curvature of the IF. The method is based on the assumption the brain is approximately centered, and initializes two reference planes 2 cm apart from the central sagittal slice of the image (Kuijf et al., 2014). A single probability distribution *p* of the intensity values in the two reference planes is created. All sagittal slices between the two reference planes are inspected, and the KL divergence is used to compute the difference *d*, between *p* and the inspected planes. Since the IF contains CSF, which presents itself as low intensity, it is expected that the difference (*d*) between the MSP and reference slices will be large (Kuijf et al., 2014). The sagittal slice that produces the largest difference *d* between the reference planes was chosen as the MSP. The estimated MSP is used to initialize the MSS, where the MSS is defined as a bicubic spline and a set of control points in a grid are placed on the MSP. These control points are adjusted in the left-right direction by using KL divergence as a cost function for optimization, by maximizing *d*, the difference between the reference planes and MSP. (Kuijf et al., 2014). A potential challenge of this approach is poor optimization in slices that contain the septum pellucidum, the membrane separating the lateral ventricles. This error results in the MSS being estimated through one of the CSF filled ventricles (Kuijf et al., 2014).

#### 2.3.1. Hausdorff Distance

The first validation metric explored is the Hausdorff distance (HD) which has been traditionally used to compare two sets of points (Olson, 1998). A smaller HD indicates better similarity between two sets. This metric is used to determine the distance or similarity between automated and manually generated midlines. The HD computes the minimum distance from a point in line segment 1 (*L*_1_) to every point in line segment 2 (*L*_2_). In this application, *L*_1_ corresponds to the ground truth and *L*_2_ the automated midline. This is repeated for every point in *L*_1_, which creates a distance vector *d*_1,2_ that contains all the minimum distances found. The distance vector, *d*_1,2_, can be found by:

(1) $$\begin{array}{l} d_{L_{1},L_{2}}\left(L_{1},L_{2}\right)=\underset{p\in L_{1}}{\text{min}}\underset{q\in L_{2}}{\text{min}}\|p-q\|. \end{array}$$

Similarly, comparing *L*_2_ to *L*_1_ the distance vector *d*_2,1_ can be computed by

(2) $$\begin{array}{l} d_{L_{2},L_{1}}\left(L_{2},L_{l}\right)=\underset{p\in L_{2}}{\text{min}}\underset{q\in L_{1}}{\text{min}}\|p-q\|. \end{array}$$

To quantify overall performance of the midline estimation, *d*_*min*_ is generated by concatenating *d*_1,2_ and *d*_2,1_ and the mean of the minimum distances is computed from to generate the mean Hausdorff distance:

(3) $$\begin{array}{l} \;d_{\text{meanHD}}\left(L_{1},L_{2}\right)=\frac{1}{N}\overset{N}{\underset{i=1}{\sum }}\left(d_{min}\left(i\right)\right) \end{array}$$

where *N* is the length of the minimum distances vector, which describes the average error between the estimated and manual midlines. This is a slight modification from the traditional HD definition that uses the maximum distance, which does not apply for midlines. For example, for a midline that has large curvature in the occipital area, a point at the top of the midline is farthest from a point near the bottom. The maximum would then yield this as high error, which is not an accurate representation of the error. Using mean HD, the average of the minimum distances is computed. The smaller the minimum distances are, the lower the average error.

#### 2.3.2. Mean Absolute Distance (MAD)

The mean absolute distance (MAD) is a pixel by pixel distance comparison of the ground truth to the estimated midline. It is an adaptation of the average z-distance, originally proposed by Ruppert et al. (2011). The z-distance was proposed to solve the problem of parallel planes, by measuring the physical distance between the estimated and ground truth MSP. It became a gold standard validation metric in the literature surrounding midline plane estimation, and has been used to validate MSP algorithms on CT and MR images (Qi et al., 2013; Rehman and Lee, 2018). Since the mid-sagittal surface can vary slice to slice based on the curvature of the IF, and is not represented by a plane, this metric was adapted to measure the physical distance between automated and annotated midlines for each slice. For a given slice, let the y-coordinates correspond to the columns, and x-coordinates corresponds to the rows. Then, the y-coordinates of the midline are used to compute the distance between each line and are averaged across cerebral slices in the volume. Let *y*_1_ be the y-coordinates of the ground truth midline and *y*_2_ y-coordinates of the estimated midline. Thus, the mean absolute distance (MAD) can be defined by:

(4) $$\begin{array}{l} MAD=\frac{1}{N}\overset{N}{\underset{i=1}{\sum }}\left|y_{1}\left(i\right)-y_{2}\left(i\right)\right| \end{array}$$

where *N* is the length of the coordinate vector. This formula is computed for each slice, and the average of cerebral slices is taken, to represent the average distance for the volume. Using the voxel spacing parameters, the average pixel distance is computed as a physical distance in *mm*. The smaller the distance, the more accurate the estimation is.

#### 2.3.3. Volume Difference (VD)

The volume difference (VD) metric is used to validate the segmented cerebral hemispheres with the difference in the volumes of the hemispheres obtained by the automated algorithm and the ground truths. The difference is computed for left and right hemispheres separately. For optimal performance the difference in volume should be 0. To compute volumes, the number of non-zero pixels are counted for each cerebral hemisphere and the voxel resolution parameters are used to compute the physical volume in mL. Let $V_{L}^{GT}$ be the ground truth volume of the left hemisphere and ${V_{L}}^{auto}$ be the automated extraction volume of the left hemisphere. The volume difference for the left hemisphere can be computed by:

(5) $$\begin{array}{l} {\Delta V}_{L}=\left|V_{L}^{GT}-V_{L}^{auto}\right|. \end{array}$$

Similarly, for the right hemisphere:

(6) $$\begin{array}{l} {\Delta V}_{R}=\left|V_{R}^{GT}-V_{R}^{auto}\right|, \end{array}$$

where $V_{R}^{GT}$ and $V_{R}^{auto}$ are the ground truth and automated volumes for the right hemisphere.

### 2.4. Midline Outlier Detection

In this section, we propose a novel metric called the volumetric asymmetry index (AI) to estimate the performance of the midline algorithm prospectively and without validation data. This can be used to automatically judge midline estimation performance in large clinical datasets, or real-time in a clinical setting. Since there is some degree of symmetry in the brain between cerebral hemispheres the difference in volume between the left and right hemispheres is expected to be small. If the difference is small, it can be assumed there are minimal (or no) errors in midline estimation. The left and right hemispheric volume is computed in mL and the AI is found by:

(7) $$\begin{array}{l} AI=\frac{|V_{L}-V_{R}|}{\left(V_{L}+V_{R}\right)} \end{array}$$

where, *V*_*L*_ and *V*_*R*_ correspond to the left and right hemispheric volume, respectively. Computing the volumetric asymmetry index on a large cohort of images gives the relative distribution of volume asymmetry in a population. Using this distribution, and a given AI computed for a prospective subject, z-score outlier analysis can be used to automatically determine the quality of the midline estimation. If the AI value is greater than three standard deviations from the mean AI value, then these outliers can be visually inspected. Any midline errors can be flagged and removed from large dataset analysis.

## 2.5. Cerebral Symmetry Analysis

Clinically, midline estimation can be used to extract biomarkers across hemispheres that can be used to explore the relationship between cognition and brain asymmetry in AD and other forms of dementia. There may be loss of gray matter, microstructural damage, increased ventricle volumes which may have a hemispheric dependence. As a proof of concept, in this work, we compute a novel hemispheric symmetry marker that investigates microstructural differences in multicenter FLAIR MRI for the CCNA dataset through local texture analysis. CCNA is used since clinical diagnosis is available for these subjects. After midline extraction, the intensity standardized images are spatially normalized to 0.35 × 0.35 × 3 mm^3^ to ensure biomarkers are comparable across subjects. Following this, the normal-appearing brain matter (NABM) is extracted through thresholding the intensity standardized images between 200 and 400 (Reiche et al., 2019). Thresholding in this range removes any CSF and white matter lesions from the symmetry analysis which aids in investigation of strictly the local changes in the NABM.

To quantify microstructural changes in the NABM, local binary patterns (LBP) are used to create texture maps. LBP is a popular texture method due to its high discriminative power and low computational expense. LBP has been used as a texture feature for dementia classification in the work by Oppedal et al. (2015). LBP detects reoccurring patterns, such as ridges and curves, which can be related to the structural integrity of the tissue. To compute the LBP texture maps, eight neighbors and a radius of 3 was used, which resulted in a 7 × 7 window. As LBP maps were generated for each slice independently, pixel-wise averaging across slices was performed to obtain a volume-wise local average of the LBP feature. This results in a single image that describes the integrity of the NABM tissue for each hemisphere.

Using the pixel-wise average texture image for the NABM, 10 different features are extracted, which are combination of first and second order histogram statistics. The first order histogram features used were mean, median, variance, skewness and kurtosis. The second order histogram features used were contrast, energy, correlation, homogeneity and entropy. Each feature *f* is computed from the respective hemisphere, and asymmetry is measured by taking the normalized difference across hemispheres, as in:

(8) $$\begin{array}{l} \Delta f=\frac{|f_{L}-f_{R}|}{\left(f_{L}+f_{R}\right)}. \end{array}$$

Statistical testing was performed using ANOVA and Tukey's Honest Significant Difference (HSD) to measure statistical differences between groups. Prior to statistical testing, the Box-Cox power transform was applied to stabilize variance and strengthen the normality of the symmetry features (Box and Cox, 1964). Statistical significance will indicate whether there are differences between brain asymmetry and cognitive groups, or diagnosis. To control for the effect of age and sex, ANCOVA analysis was performed. This allows the relationship between the symmetry markers and diagnostic labels to be analyzed independently.

## 3. Results

In this section, results are visualized, followed by quantitative performance evaluation on the midline validation set, outlier rejection analysis on the entire dataset and symmetry analysis on CCNA. For midline estimation accuracy, the mean performance metrics are computed over all 75 ground truth volumes. To test the reliability and consistency of the method, validation metrics are compared as a function dataset (CCNA, ADNI, CAIN), scanner vendor (GE, Philips and Siemens), and pathology, by measuring the CSF load in the validation cases. Midline detection performance is compared to the two previous works by Bergo and Kujif. To investigate performance on large clinical datasets, the midline is extracted over all datasets, which comprises 5,360 volumes (roughly 275,000 image slices) from over 80 centers worldwide. The volume asymmetry index is computed and the outlier volumes are visually inspected. Using the remaining volumes, asymmetry biomarkers measured from CCNA will be analyzed and correlated with diagnosis to show proof of concept and clinical feasibility of the work.

### 3.1. Midline Visualizations

**Figure 3:**
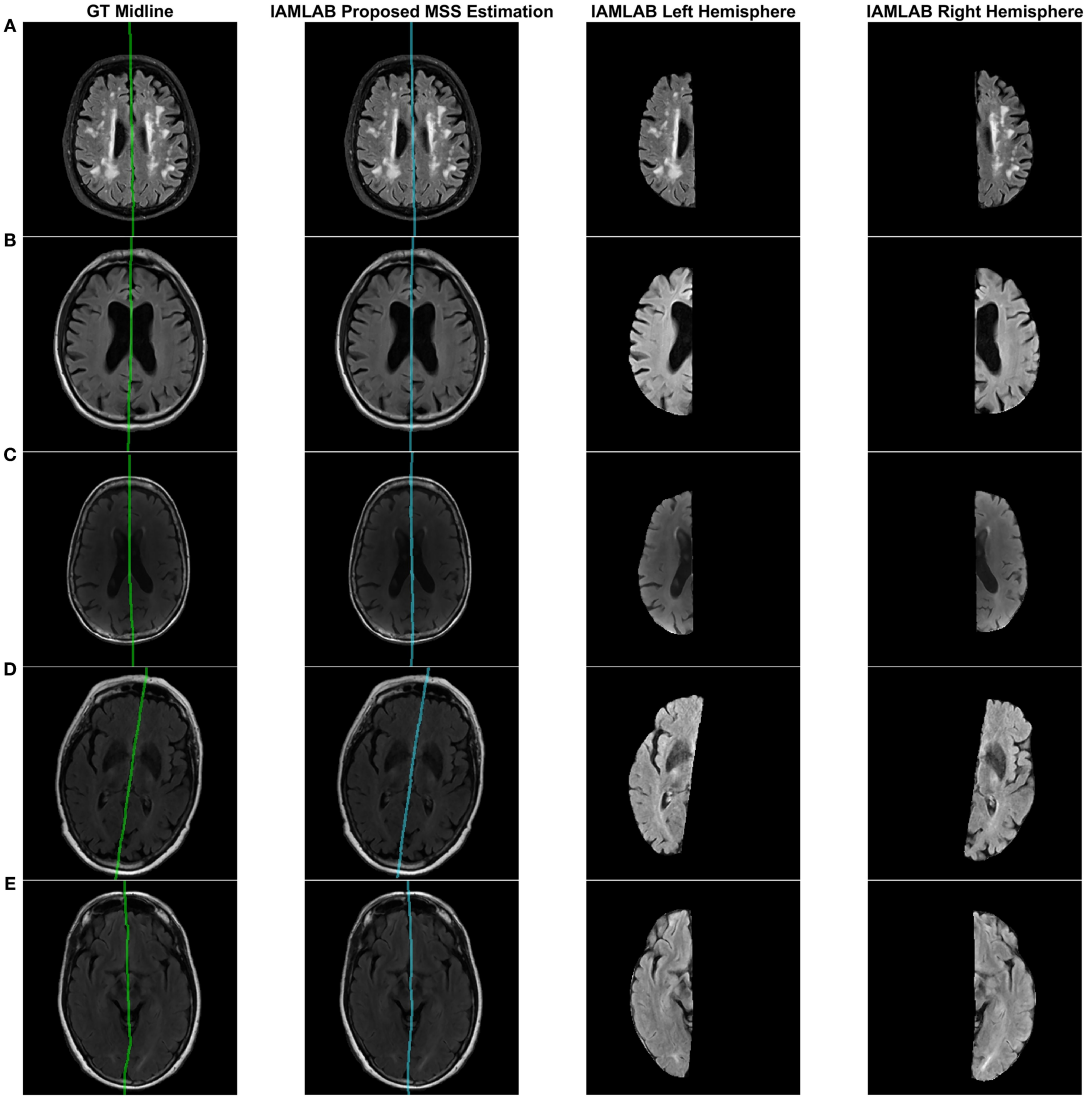
Visualizing midline estimations and extracted hemispheres of various cases using the proposed method. From left to right: ground truth midline, estimated midline, left hemisphere, right hemisphere. The cases shown are: **(A)** High lesion load, **(B)** Enlarged ventricles, **(C)** Dark contrast, **(D)** Head angled, and **(E)** Curved LF.

Figure 3 shows the results of the proposed midline estimation method on several cases. The first column displays the ground truth midlines, the second column is the estimated midline and the last two columns contain the left and right hemispheric segmentations. For images with dark contrast, large ventricles or heavy lesion loads the tool robustly estimates the midline. There is also an example with extreme curvature and off-center head angle, and in both cases, the midline is accurately estimated as well. Hemispheric separation clearly shows the two hemispheres contain tissue only from the respective hemisphere.

**Figure 4:**
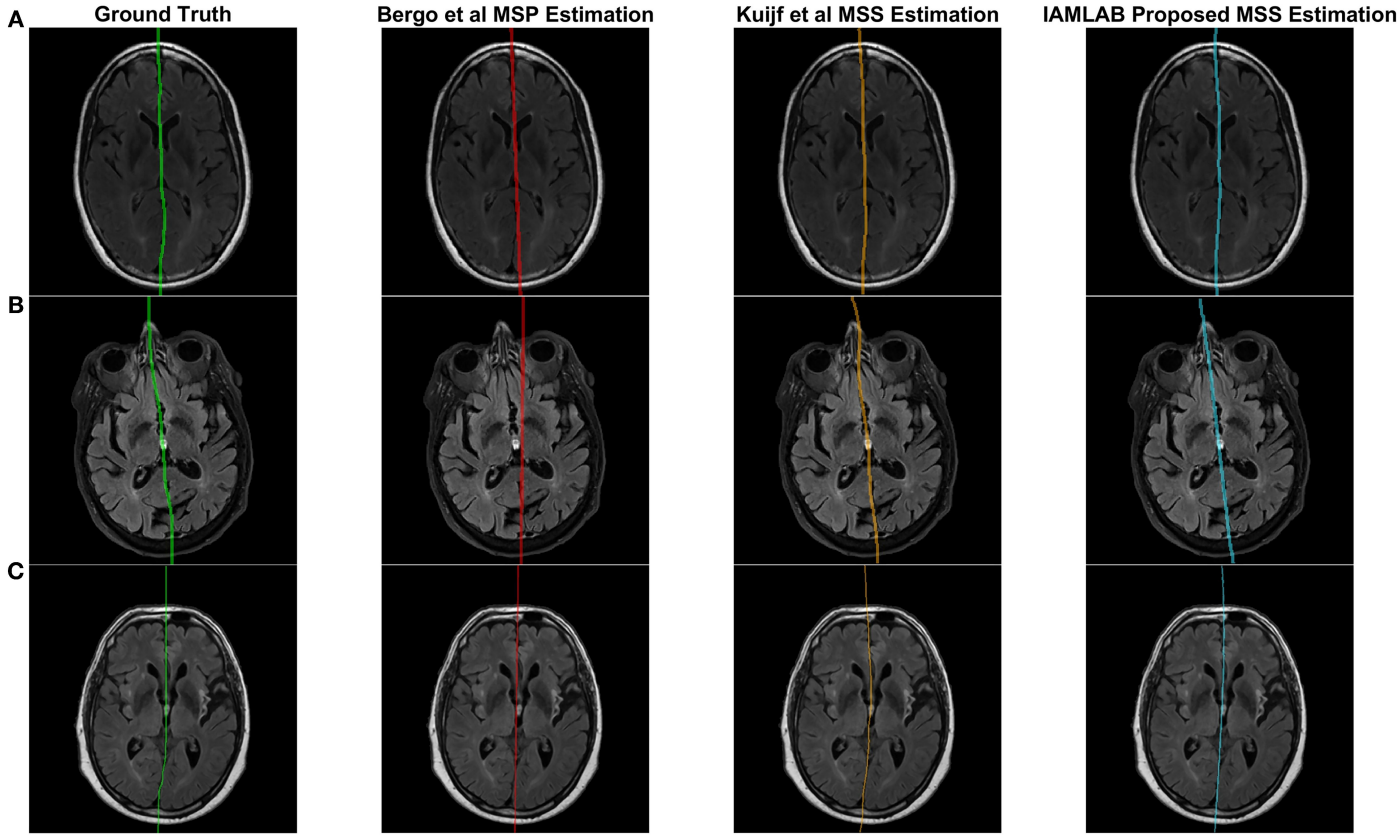
Sample midline estimation results for the proposed method compared to the state-of-the-art. **(A)** ADNI. **(B)** CAIN. **(C)** CCNA.

**Figure 5:**
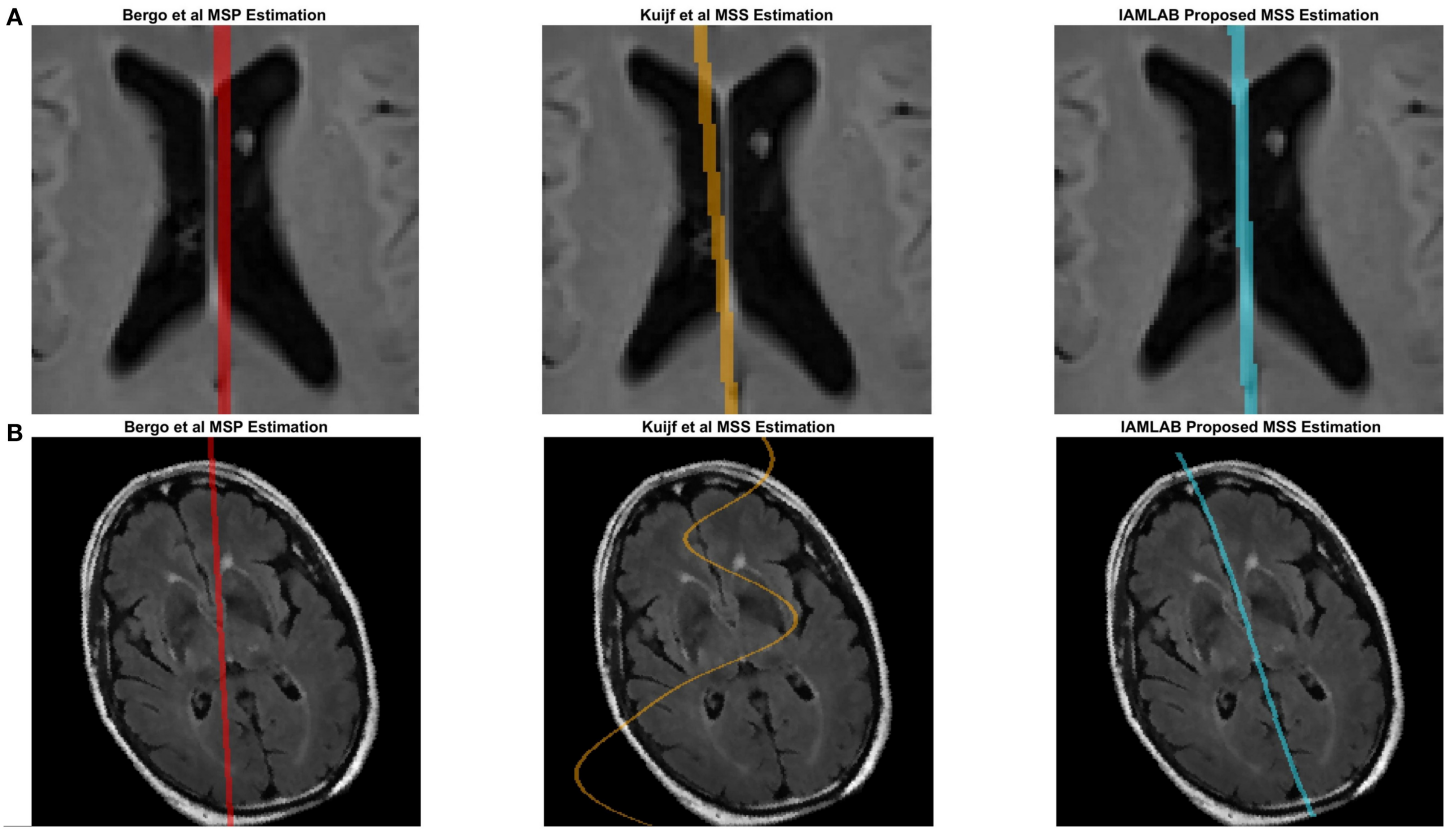
Challenging examples for midline estimation. **(A)** Septum pellucidum. **(B)** Head rotation.

Figure 4 contains the estimated midline for the proposed method alongside the two competing methods (Bergo and Kujif) for ADNI, CAIN, and CCNA. As shown, the proposed method carefully delineates the interhemispheric fissure and adapts to the curvature across the datasets. The midline plane estimation method (Bergo) fails to estimate the curvature of the IF due to the planar nature of the method. Kuijf's method appears to track the IF, but in Figure 5A, the MSS is estimated through one of the CSF-filled ventricles. During optimization, the cost function estimates the spline through the ventricles and avoids the septum pellucidum, which is likely the reason for this. Similarly, the Bergo et al. method finds the minimum intensity score inside the ventricles and since the septum pellucidum contains high intensities, this method cannot accurately estimate the midline in this region. The proposed method over comes these challenges through multiple features and intensity inversion during control point estimation, resulting in accurate demarcation of the midline through the septum pellucidum and for curved interhemispheric fissures.

To demonstrate the benefits of the angle correction step, and to further demonstrate the robustness of the approach, an additional experiment was conducted and the results are shown in Figure 5B. In this experiment a FLAIR volume was rotated by 20°, and the midline estimation algorithms were employed. The proposed method highlights the midline more robustly than the comparison methods.

### 3.2. Accuracy

**Figure 6:**
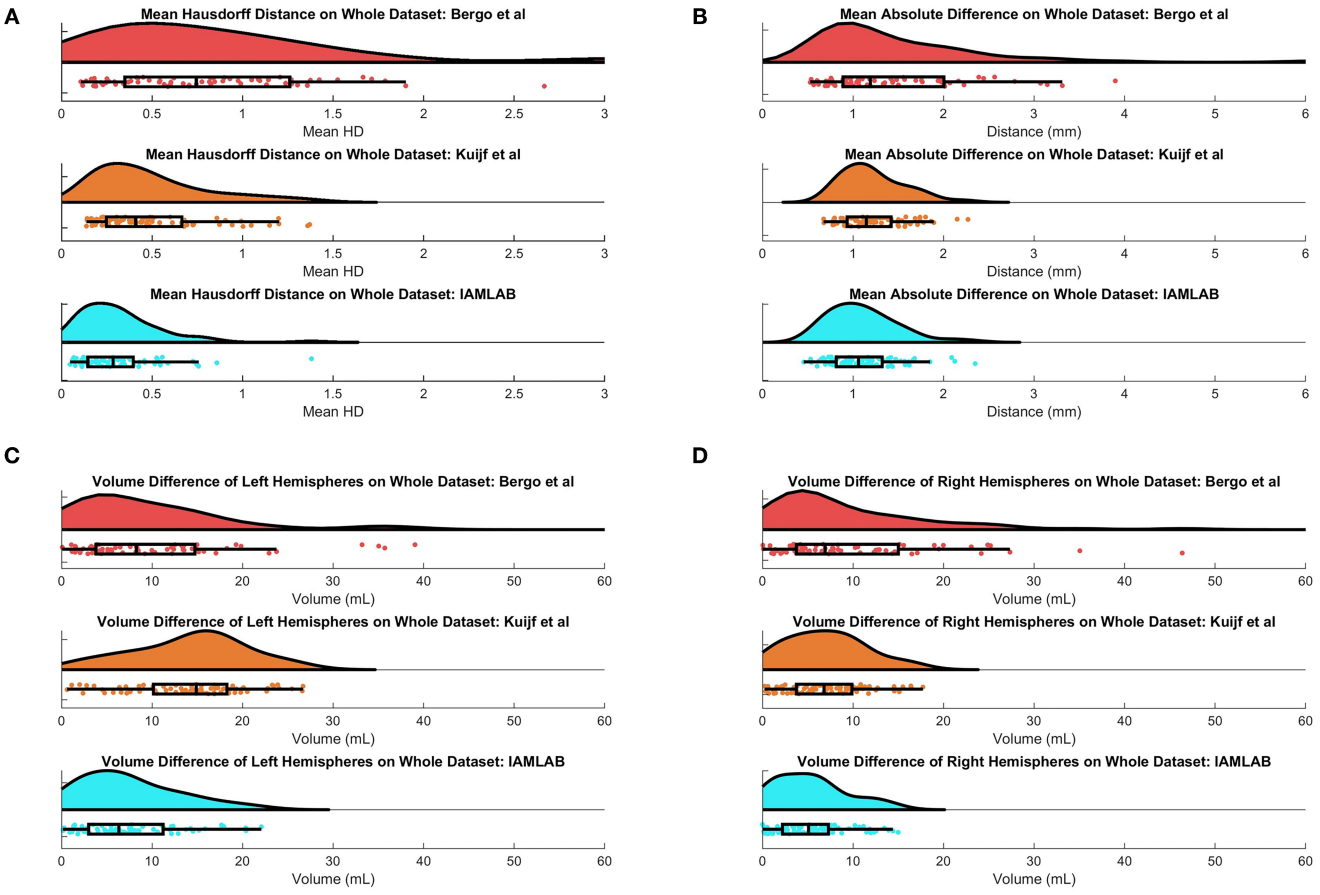
Raincloud plots of results for **(A)** Mean HD, **(B)** MAD, **(C)** Left Volume Difference Δ*V*_*L*_, and **(D)** Right Volume Difference Δ*V*_*R*_ for entire validation set (75 volumes from CAIN, ADNI, and CCNA).

In this section, midline estimation accuracy is reported by the mean Hausdorff distance (HD), volume difference (VD) and mean absolute difference (MAD) of both hemispheres. The estimated midline is compared to the ground truth delineations in the midline validation data (75 ground truth volumes from CAIN, ADNI, and CCNA). The mean and standard deviation for each metric is summarized in Table 3 and raincloud plots for each metric are shown in Figure 6 to visualize the distributions (Allen et al., 2019). The proposed method has the lowest mean Hausdorff distance (HD) distance (and standard deviation) with a much more compact distribution, indicating the proposed method is more accurately estimating the midline across multicenter datasets as compared to the competing methods. Mean VD across both L and R hemispheres, and MAD are also the best for the proposed method. This is likely due to both the intensity standardization framework and robust feature measurements that can handle IF curvature. The next best performer was the method by Kujif et al., followed by the traditional midline plane estimation technique. The outliers in the Bergo et al. method may be attributed to the planar nature of the method.

**Table 3:** Mean (± std) validation metric for all methods over the entire validation dataset.

	**Bergo et al**.	**Kuijf et al**.	**IAMLAB**
Mean HD	1.20 ± 1.73	0.49 ± 0.31	**0.32 ± 0.23**
MAD (mm)	2.13 ± 2.82	1.22 ± **0.35**	**1.10** ± 0.38
Δ*V*_*L*_ (ml)	15.97 ± 26.51	14.18 ± 6.31	**7.52 ± 5.40**
Δ*V*_*R*_ (ml)	15.57 ± 26.63	14.18 ± 6.31	**5.35 ± 3.97**

*Bold is best*.

### 3.3. Reliability and Consistency

**Figure 7:**
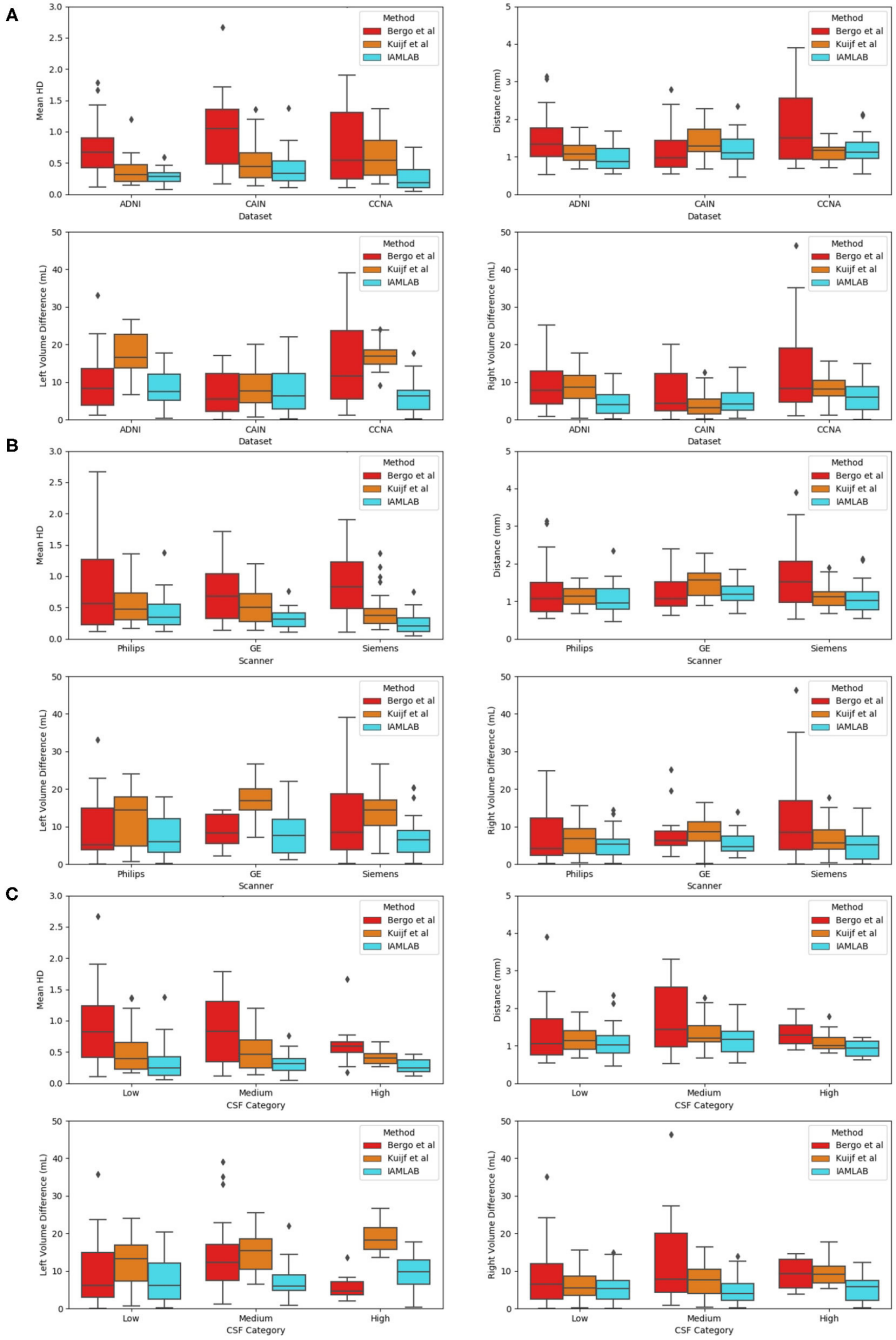
Validation metrics as a function as a function of **(A)** dataset, **(B)** scanner, and **(C)** CSF load.

To investigate the reliability and consistency of the proposed method, validation metrics are compared as a function of dataset, scanner vendor, and pathology in this subsection. The validation results for all groups are shown in Figure 7 and summarized in Table 4.

**Table 4:** Mean (± std) of mean HD and mean volume difference for each dataset, scanner type and pathology level.

	**Mean HD**			**Δ V (mL)**		
	**Bergo et al**.	**Kuijf et al**.	**IAMLAB**	**Bergo et al**.	**Kuijf et al**.	**IAMLAB**
ADNI	0.76 ± 0.44	0.37 ± 0.22	**0.28 ± 0.13**	9.83 ± 6.94	13.08 ± 5.00	**6.16 ± 3.19**
CAIN	1.51 ± 2.01	0.51 ± 0.32	**0.41 ± 0.29**	14.84 ± 32.00	**6.25 ± 4.34**	6.82 ± 5.06
CCNA	1.34 ± 2.15	0.59 ± 0.34	**0.26 ± 0.21**	22.64 ± 31.55	12.58 ± 3.32	**6.33 ± 3.27**
GE	1.29 ± 2.34	0.56 ± 0.34	**0.34 ± 0.17**	17.13 ± 35.91	13.03 ± 4.84	**7.10 ± 4.22**
Philips	0.86 ± 0.83	0.53 ± 0.34	**0.43 ± 0.30**	8.60 ± 7.78	9.62 ± 6.38	**6.30 ± 4.33**
Siemens	1.35 ± 1.81	0.43 ± 0.28	**0.25 ± 0.17**	19.16 ± 28.28	10.19 ± 4.52	**6.23 ± 3.57**
Low	1.28 ± 1.82	0.50 ± 0.34	**0.33 ± 0.28**	14.77 ± 26.85	9.20 ± 5.25	**6.56 ± 4.36**
Medium	1.25 ± 1.83	0.49 ± 0.31	**0.32 ± 0.17**	19.35 ± 29.28	11.41 ± 4.77	**5.98 ± 3.11**
High	0.66 ± 0.45	0.42 ± 0.13	**0.27 ± 0.12**	7.56 ± **3.28**	14.62 ± 4.82	**7.51** ± 4.39

*Bold is best. Low, medium, and high refer to CSF loads: <150 mL, 150–250 mL, >250 mL*.

#### 3.3.1. Results as a Function of Dataset

Three datasets are used to analyze performance: ADNI, which is an AD dataset, CAIN which is a vascular disease dataset and CCNA which is a dementia dataset. Testing the midline estimation methods over each dataset can be used to gauge robustness across diseases. As discussed in section 2.1, there are a variety of vascular and dementia diseases in the midline validation dataset. In Figure 7A, the mean HD, mean absolute difference (MAD) and volume difference (VD) metrics are shown for each dataset and method and summarized in Table 4. The proposed technique shows lowest mean error (HD and VD) and standard deviation over most datasets. The proposed technique yields the lowest mean and standard deviation for mean HD in the ADNI, CAIN, and CCNA groups. Kuijf et al. method yields a lower mean and standard deviation for the CAIN dataset, followed closely by the proposed technique. For CCNA and ADNI, the proposed method yields the lowest mean VD and standard deviation. The lowest performance is from the Bergo et al., method due to head angle errors or due to the planar nature of the approach.

#### 3.3.2. Results as a Function of Scanner Vendor

The datasets were acquired using one of the three scanner vendors: Philips, GE, or Siemens. This causes variability in the data as each scanner vendor has varying software, post-processing techniques, MR acquisition parameters and hardware components. In Figure 7B, mean HD, MAD, and volume difference metrics are grouped per scanner vendor for each compared method. The proposed technique shows lower error over each scanner and a more consistent distribution across the scanner vendors. The mean and standard deviation for both metrics, mean HD and mean VD, is lowest for the proposed technique across all scanners as shown in Table 4. The proposed method produces minimal error across GE, Philips and Siemens scanners, and has similar performance across scanners which highlights the clinical feasibility and reliability of the method.

#### 3.3.3. Results as a Function of Pathology

To determine if the proposed work is robust to the level of disease, midline estimation performance is analyzed as a function of CSF load to determine reliability across varying disease levels. A common characteristic of neurodegenerative diseases is increased ventricular volume and atrophy (Ott et al., 2010). Therefore, to quantify disease burden in AD, vascular disease or dementia subjects, the CSF load in the ventricles and subarachnoid spaces is measured for each imaging volume. CSF load is chosen as a disease characteristic since large ventricles, or high amounts of atrophy can create challenges in midline segmentation algorithms. CSF load is computed in the intensity standardized FLAIR MRI by determining by applying a threshold of 200 to extract a CSF mask from the standardized brain over the entire dataset (Reiche et al., 2019). Using the number of non-zero pixels and the voxel spacing parameters, CSF volume in mL was found.

Plots of each validation metric against CSF load were used to visualize trends in performance with increasing pathology. In Figure 7C, the mean HD, MAD and volume difference, the proposed technique had the lowest average error over all loads (low, medium, and high). For both mean HD and MAD, the average error is the lowest in all three CSF categories. This validates the ability of the proposed method to estimate the midline correctly when the lateral ventricles increase in neurodegenerative cases. This is also illustrated in Figure 3B. When looking at the volume difference metric for the Bergo et al. method, it is affected by low and medium CSF load cases. This method relies is based on minimizing a global intensity score in the plane, and thus with less CSF, the global score will struggle in finding an optimal plane. The proposed method performs feature optimization locally, which improves the estimation of IF in low to medium CSF load cases. The Kuijf et al. method struggles the most with high CSF load cases, due to the optimization algorithm estimating into the lateral ventricles. Overall, the proposed method yields the lowest average volume difference for low, medium and high CSF loads.

### 3.4. Midline Outlier Detection

In this section, the proposed midline algorithm is computed for each of the 5,360 volumes from the entire dataset (roughly 275,000 image slices for ADNI, CAIN, and CCNA combined) and the asymmetry index (AI) is used to automatically assess midline estimation performance without ground truths. The AI measures the difference in volume between hemispheres for every volume in the dataset, and the distribution of the AI values were retained for z-score outlier analysis. Volumes with extreme AI values are flagged for visual analysis to verify hemispheric segmentation visually. In a clinical setting, this tool can be used when ground truth data is unavailable or infeasible to obtain. For research applications and large-scale analysis, the AI can be used to determine sub-optimal segmentation results (which can therefore be excluded from biomarker studies). The AI outlier detection method drastically reduces the number of cases to visually inspect in large datasets and indirectly measures hemispheric segmentation performance without ground truth. Out of a total of 5,547 volumes, only 53 volumes were detected as outliers (<1%) using z-score analysis, with 33 out of 4,100 in ADNI (0.8%), 10 out of 871 in CAIN (1.1%), and 10 out of 380 volumes CCNA (2.6%). The 53 outliers were then visually inspected to verify the outliers.

**Figure 8:**
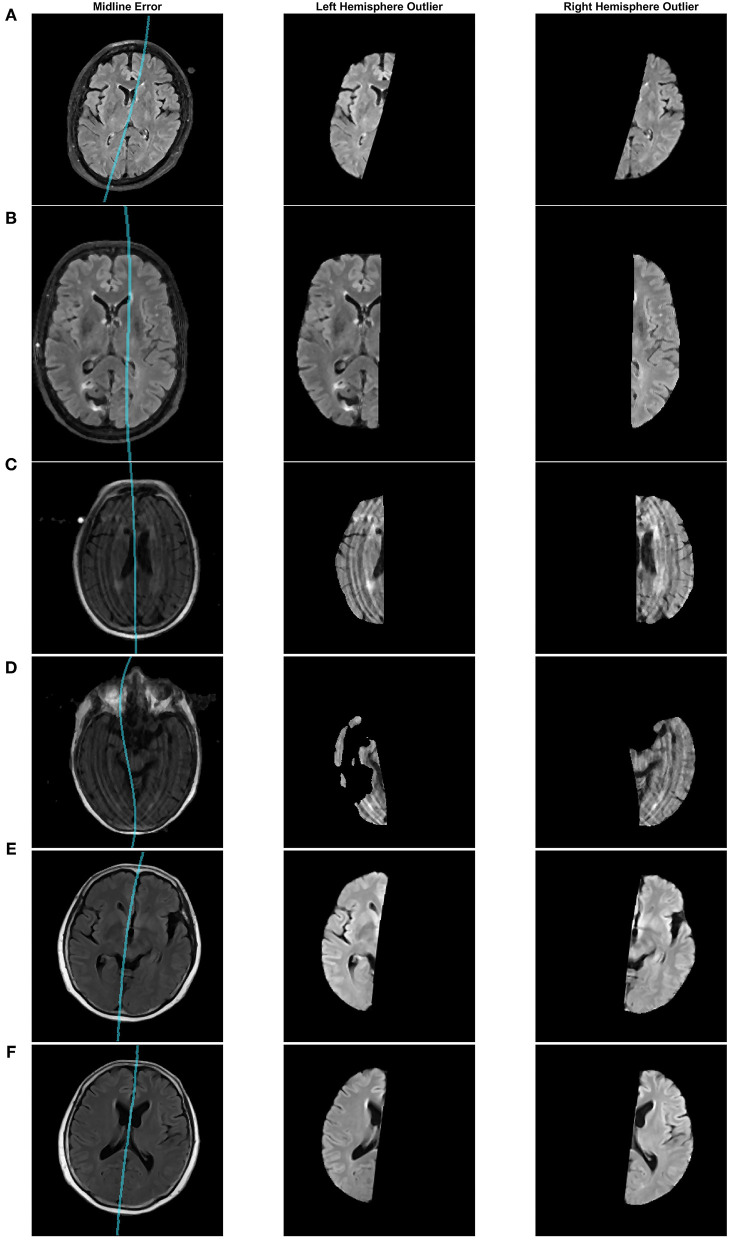
Midline estimation errors detected using the asymmetry index outlier detection method. Cases from **(A–F)**: CCNA, CAIN, ADNI, ADNI, ADNI, ADNI.

Some of the detected outliers are shown in Figure 8. Figure 8A is a case from the CCNA dataset that was found to be estimated incorrectly. Upon further inspection, this error was caused by incorrect estimation of the head angle during preprocessing. The head angle was measured to be -15 degrees (which visually can be seen to be incorrect since the brain is not angled). The cross-correlation score fails here because of the shape of the head. When the shape is circular, cross correlation score reaches a maximum at various rotations of θ. To make this more robust in the future perhaps an edge map of the brain could be incorporated into the cross correlation analysis (Liu et al., 2001). In (B), the brain is not centered which misaligns the detection of the 2 cm rectangular ROI that should contain the midline. In (C), there are large motion artifacts, which although creates some inaccuracies, overall, the proposed technique still manages to approximate the midline, given the poor quality of the image. Another poor quality case, in (D), has found an issue with the brain segmentation mask due to missing tissue. These outliers, whether it be from segmentation error or poor image quality, can be removed prior to clinical analysis.

After visual inspection of the 53 outliers, only one was seen to be a case that can be accepted and used in the clinical analysis. Figures 8E,F, shows two different slices from this case in the ADNI dataset. In certain slices, the midline estimation is slightly off and not optimal, as seen in (E). For the rest of the volume and majority of the later cerebral slices, the midline estimation was found to be accurate. Due to the slight underestimation of the midline on a few slices, this case was found to have a z-score value of 3.154, which is just greater than the z-score cut off of 3, making this an outlier by definition. This highlights the sensitivity of the AI outlier method to slice differences in volumes as well.

### 3.5. Clinical Symmetry Analysis

**Figure 9:**
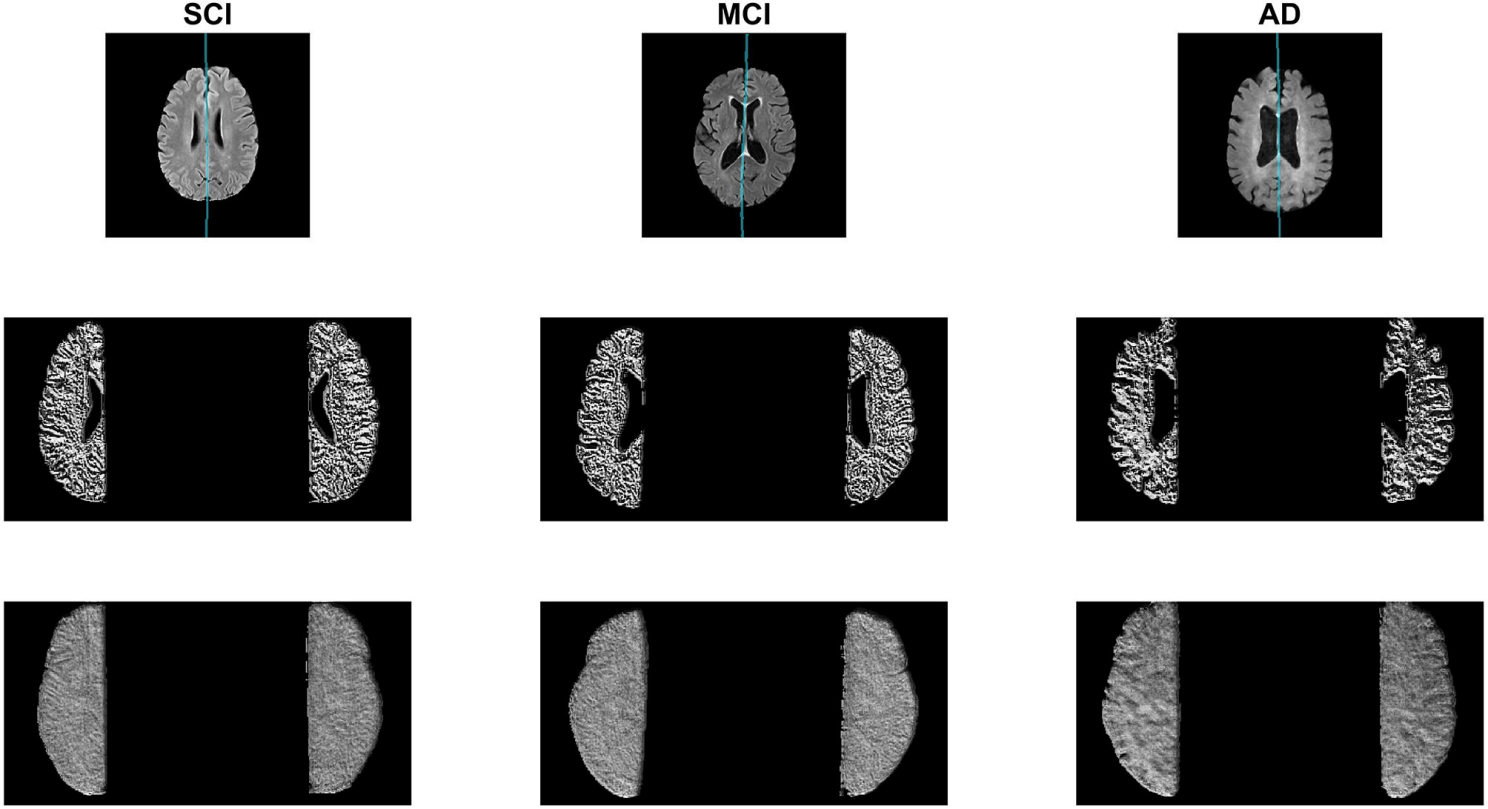
Sample AD, MCI, and SCI slice **(Top)** with corresponding LBP slice **(Middle)** and LBP pixel-wise average **(Bottom)**.

In this subsection, the midline is extracted from the SCI, MCI, and AD labeled CCNA volumes and the proposed asymmetry biomarkers in FLAIR are extracted and analyzed to demonstrate proof-of-concept. First the midline is extracted from the whole volume and cerebral hemispheres are extracted. To measure biomarkers in the normal-appearing brain matter (NABM) thresholds are applied to the intensity standardized data to strip out the CSF and lesions (Reiche et al., 2019). The hemispheres of the NABM are segmented and the LBP is computed on a per-slice basis on each hemisphere separately. The pixel-wise average of the LPB feature map is taken across the volume for each hemisphere, and statistical values are computed per hemisphere. To quantify differences in hemispheric properties, asymmetry is measured by taking the normalized difference across hemispheres for each statistical feature. See Figure 9 for example images with the detected midline, central slice of the LBP feature map and the pixel-wise average for a subject with SCI, MCI and AD. Considering the feature maps, there are visual differences in texture across diseases. As cognitive impairment increases from SCI, to MCI and to AD, the roughness of the NABM increases and the ridges and curves are much larger which could indicate a breakdown in NABM integrity.

**Figure 10:**
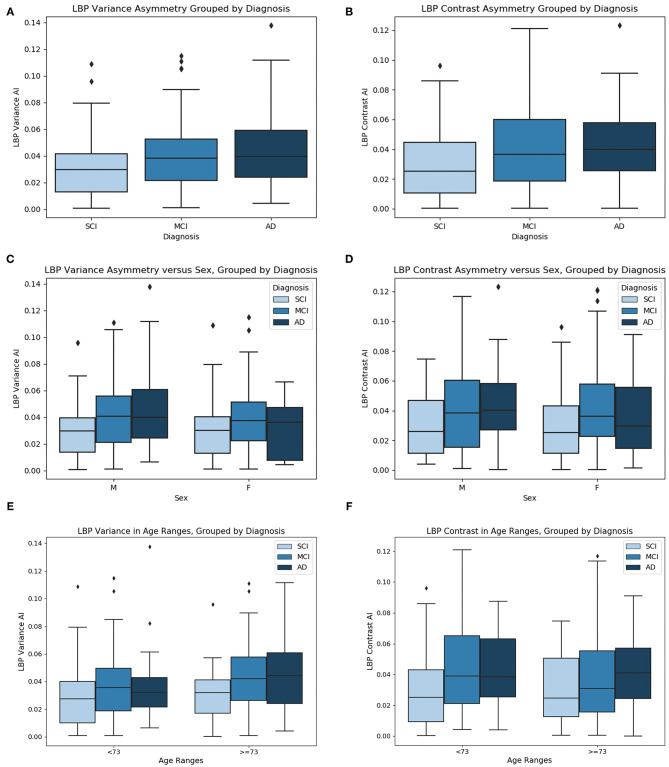
**(A)** LBP variance asymmetry, **(B)** LBP contrast asymmetry grouped by diagnosis, **(C)** LBP variance asymmetry vs. sex, grouped by diagnosis, **(D)** LBP contrast asymmetry vs. sex, grouped by diagnosis, **(E)** LBP variance asymmetry vs. age grouped by diagnosis, and **(F)** LBP contrast asymmetry vs. age, grouped by diagnosis. Age ranges split by median age.

Prior to comparing biomarker means across groups, outlier cases are removed. After outlier removal there are 47 SCI cases, 96 MCI cases, and 40 AD cases. See Figures 10A,B for the mean LBP variance and contrast asymmetry features plotted as a function of cognitive diagnosis. The level of asymmetry measured by LBP variance and contrast increases with worse cognitive outcome, indicating there is a difference in the NABM texture across hemispheres for each disease level.

One-way ANOVA was used to test if the mean values of the symmetry biomarkers were significantly different between disease groups SCI, MCI, and AD. In Table 5, ANOVA analysis found the means of the symmetry biomarkers to be different across disease groups, for both LBP variance and contrast symmetry features. To further investigate the sources of the differences, Tukey's HSD was used for *post-hoc* analysis in Table 5. *Post-hoc* testing revealed significant differences between MCI and SCI groups for both variance and contrast asymmetry features with a mean difference of 0.053 (*p* = 0.005) and 0.068 (*p* = 0.003), respectively. Moreover, significance was found between AD and SCI groups with a difference in means = 0.069 (*p* = 0.005) and 0.067 (*p* = 0.032) for variance and contrast, respectively. Therefore, textural symmetry biomarkers from the NABM can be used to distinguish between these disease groups. No statistical significance was found between AD and MCI groups.

**Table 5:** One-way ANOVA and ANCOVA tests with *post-hoc* analysis for each asymmetry biomarker.

**Test**	**Feature**	**Source**	**F**	**Pr > *F*_*crit*_**	**SCI vs. MCI**	**MCI vs. AD**	**SCI vs. AD**
ANOVA + Tukey's HSD	LBP variance	Diagnosis	**6.404**	**0.002**	**0.005**	0.702	**0.006**
	LBP contrast	Diagnosis	**5.894**	**0.003**	**0.003**	0.9	**0.032**
ANCOVA (Age) + *post-hoc*	LBP variance	Diagnosis	**4.070**	**0.018**	**0.027**	0.95	**0.04**
		Age	3.631	0.058	–	–	–
	LBP contrast	Diagnosis	**6.551**	**0.002**	**0.002**	0.999	**0.018**
		Age	1.322	0.251	–	–	–
ANCOVA (Sex) + *post-hoc*	LBP variance	Diagnosis	**4.560**	**0.011**	**0.018**	0.81	**0.025**
		Sex	1.316	0.252	–	–	–
	LBP contrast	Diagnosis	**5.677**	**0.004**	**0.003**	0.999	**0.033**
		Sex	0.112	0.739	–	–	–

*Bold values indicate statistical significance*.

To investigate biomarker differences across age and sex, the variance, and contrast asymmetry features are analyzed further. Figures 10C,D contains the distribution of the biomarkers as a function of sex, and Figures 10E,F shows the biomarkers as a function of age, using the median age as a cutoff for group comparison. The same trend observed in Figures 10A,B is seen, where the biomarkers across disease groups for sex or age have increasing asymmetry measured by LBP variance and contrast from SCI, to MCI and to AD. To validate the findings of Figures 10C–F, ANCOVA testing was completed to statistically analyze the difference in biomarker means while first controlling for age, and next controlling for sex. These results are summarized in Table 5. When controlling for age, the relationship between the asymmetry features and diagnosis remained statistically significant. Performing *post-hoc* analysis with the effect of age removed revealed significant differences between MCI and SCI groups for both variance and contrast asymmetry features (*p* = 0.027 and *p* = 0.002, respectively). Moreover, significant differences between AD and SCI groups are found after ANCOVA for both variance and contrast asymmetry features (*p* = 0.04 and *p* = 0.018, respectively). Similar to ANOVA, no significant differences were found between MCI and AD groups. Although the features remain significant, the *p*-values marginally increased, implying that age has some effect on textural asymmetry. This is expected as microstructural integrity, GM, and WM loss is found to increase with age (Ge et al., 2002). Although there is an age affect, the proposed biomarkers remain significant across disease levels. When considering sex as a co-variate, it was not found to be statistically significant for both LBP variance and contrast indicating that these biomarkers are not influenced by sex. Performing *post-hoc* analysis with the effect of sex removed revealed significant differences between MCI and SCI groups for both variance and contrast asymmetry features (*p* = 0.0018 and *p* = 0.003, respectively). Moreover, significant differences between AD and SCI groups are found after applying ANCOVA with sex removed, for both variance and contrast asymmetry features (*p* = 0.025 and *p* = 0.033, respectively). No significant differences were found between MCI and AD groups.

## 4. Discussion

The proposed midline estimation technique is completely unsupervised, can adapt to curvature in the interhemispheric fissure (IF), does not require an initial plane estimate preprocessing step, estimates the midline for each slice individually for improved accuracy, and the method can robustly estimate the midline in the septum pellucidum. In terms of accuracy over the entire validation dataset of 75 FLAIR MRI volumes from 38 centers in ADNI, CAIN, and CCNA, the method yielded the lowest average error for the mean Hausdorff distance (HD), mean absolute distance (MAD) and volume difference metrics compared to two other previous works. When analyzing the reliability and consistency of the method, the performance is more consistent across datasets, scanner vendors and CSF load compared to the other methods demonstrating the ability of the proposed midline detection algorithm to effectively operate in diverse multicenter and multi-disease FLAIR MRI datasets. Performance on large datasets and automated outlier detection highlights the clinical utility of the proposed method and ability to detect midline detection inaccuracies automatically and on-the-fly. Asymmetry biomarkers that quantify the structural integrity of the normal-appearing brain matter (NABM) show significant differences between subjects with different cognitive diagnoses and provide the opportunity for larger cerebral hemisphere symmetry analysis studies in the future.

An important characteristic of the proposed method is the ability to estimate irregularities and curvature within the interhemispheric fissure. In the method by Bergo et al. (2008), irregularities in and the non-planar nature of the fissure is known to create algorithm inaccuracies and this was also seen in our experiments for the method implemented on FLAIR MRI. The method by Kuijf et al., has challenges when large head angles are present (see Figure 5) and there was more volume difference error in the ADNI and CCNA datasets. ADNI and CCNA are dementia datasets, and a common characteristic of the disease is increased ventricular size. Thus, the Kuijf et al. method may be over-estimating the midline into the lateral ventricles, which could be due to the septum pellucidum. The proposed method is also more robust to head angle variations through the use of the head angle correction step.

With the rise of deep learning in medical image segmentation, mid-sagittal surface estimation could potentially be improved upon through these techniques. A recent paper by Pisov et al. (2019) uses convolutional neural networks (CNNs) for brain midline shift (MLS) detection. They introduced a novel deep learning based approach for MLS detection, which exploits task-specific structural knowledge. The work utilizes a two-headed CNN with shared input layers, where one head is tasked with the segmentation via UNet, and the other head predicts the slices which contain MLS (Pisov et al., 2019).

In this work, preliminary clinical analysis highlighted the relationship between dementia diagnosis and asymmetry of the NABM microstructure in multicenter FLAIR MRI. It is seen that textural asymmetry increases as dementia progress from SCI to AD. This can possibly be attributed to an overall decrease in the structural integrity of the tissue since the texture feature quantifies the changes in intensity of the NABM. In regions with higher variance and contrast asymmetry, there may be more tissue degeneration in the GM and WM for a particular hemisphere. These findings are analogous to previous asymmetry studies in AD and dementia. Yang et al. used diffusion tensor tractography to construct hemispheric brain WM networks (Yang et al., 2017). They found hemispheric brain WM networks showed an aberrant rightward asymmetry in AD, but not in the early phases of MCI (Yang et al., 2017). Through voxel-based morphometry of T1-weighted MRI, Derflinger et al. (2011) found brain atrophy in AD to be asymmetric. They also report that performance of language-based neuropshycological tests are correlated with the lateralization of GM loss in the left hemisphere in AD and MCI patients (Derflinger et al., 2011). Lateralization (left vs. right asymmetry) of microstructural changes and the relationship to texture and cognitive status in FLAIR MRI will be explored further in future studies.

From all the features extracted from the 2D LPB feature map, the variance and contrast were shown to be statistically significant features to differentiate between AD—SCI, and MCI—SCI groups. No statistical significance was found between AD and MCI groups. This could be due to class imbalance between AD and MCI, or could be due to the over-generalization of the MCI diagnosis, which captures a wide range (of subjective) memory complaints and cognitive conditions. A review on the current research of MCI found that MCI has been modified from a memory disorder to include other types of cognitive concerns and impairments that describe other forms of dementia, not just progression to AD (Petersen, 2009).

In future work, additional asymmetry biomarkers will be designed and applied on more datasets and compared to additional clinical variables. Clinical variables, such as medical history, cognitive scores, and vascular disease risk factors will be investigated. Given that FLAIR MRI is the leading modality for the investigation of cerebrovascular disease, we are excited to explore this in future works.

## 5. Conclusion

Through the combination of shape and symmetry based approaches, an automated midsagittal surface estimation algorithm was designed to robustly delineate the curvature of the interhemispheric fissure (IF). It is completely unsupervised, and extracts the midline accurately on a per slice basis. Performance was compared to two state-of-the-art methods for midline estimation and the proposed method yielded the lowest average error over 75 volumes from 38 centers, acquired from GE, Siemens and Philips scanners. Performance of the proposed algorithm was also shown to be more consistent in multi-center, multi-scanner and multi-pathology datasets, and more reliable in varying levels of CSF pathology as compared to the other approaches. The midline of 5,360 FLAIR MRI volumes from 86 international centers were extracted and a novel automated asymmetry index was defined to automatically detect outliers that could be related to poor segmentations. From the 5,360 volumes <1% were detected as outliers which were easily inspected manually. Finally, clinical utility of the method was shown as asymmetry features that quantified microstructural differences in the normal-appearing brain matter (NABM) across hemispheres were shown to differentiate between cognitive diagnosis. In future work, these tools will be applied on larger datasets and correlated to clinical variables to discover relationships between brain asymmetry and neurodegenerative diseases.

## Data Availability Statement

Publicly available datasets were analyzed in this study. This data can be found at: ADNI—http://adni.loni.usc.edu/methods/mri-tool/standardized-mri-data-sets/.

## Author Contributions

AG and AK designed the proposed method and performed experiments. AG, AK, and AM wrote the manuscript. AM assisted with the clinical aspects of the work. All authors discussed the results and reviewed the final version of the manuscript.

## Conflict of Interest

The authors declare that the research was conducted in the absence of any commercial or financial relationships that could be construed as a potential conflict of interest.
